# Association of Height and Prevalence of Kidney Stones

**DOI:** 10.7759/cureus.32919

**Published:** 2022-12-25

**Authors:** Marc Ganz, Christopher Alessandro, Menachem Jacobs, Daniel Miller, Jonathan Diah, Bethany R Desroches, John M Shields

**Affiliations:** 1 Public Health Sciences, State University of New York Downstate Health Sciences University, New York City, USA; 2 Internal Medicine, Icahn School of Medicine at Mount Sinai, Queens Hospital Center, New York City, USA; 3 Urology, State University of New York Downstate Health Sciences University, New York City, USA; 4 Urology, Kings County Hospital Center, New York City, USA

**Keywords:** nephrology, urology, public health, kidney stones, nephrolithiasis

## Abstract

Introduction and objectives

Nephrolithiasis is universally understood to be a multifactorial disease resulting from genetic and environmental factors including gender, diet, calcium, and uric acid excretion. Notably, several of these factors may be related to body habitus. Because men are more likely to develop kidney stones and on average have a larger body size, height may be an important risk factor for stone formation. Several studies have demonstrated that short adult stature is associated with numerous conditions such as hypertension, hypercholesterolemia, and cardiovascular diseases. However, other studies have demonstrated otherwise. Additionally, stones have been shown to be correlated with a high body mass index (BMI). This is likely due to dietary factors. Although height is a component of BMI, there is minimal literature regarding the relationship of height to stone prevalence adjusting for weight.

Methods

We aimed to examine whether short adult height is associated with the development of kidney stones using a population-based cohort of the National Center for Health Statistics. Data was gathered from National Health and Nutrition Examination Surveys (NHANES) “Kidney Conditions - Urology” and “Weight History” questionnaire datasets from March 2017 to March 2020 along with demographic data. Logistic regression analysis was used to determine an association between current self-reported height (inches) and if the participant has ever had kidney stones, controlling for weight, gender, age, race, educational level, and marital status.

Results

We found that those who were shorter had higher odds of reporting a history of stones (OR: 1.017; 95%CI: 1.005-1.028). This association was found after controlling for covariates such as age, gender, race, education, and weight. In addition, the male gender and Hispanic race had higher odds of reporting a history of stones (OR: 1.43 and 1.073, respectively).

Conclusion

Our results suggest that short height is related to the prevalence of kidney stones independent of weight, age, gender, and race. This supports previous literature indicating height to be a component of renal disease.

## Introduction

In recent years, the prevalence of kidney stones has risen in both developed and underdeveloped countries [1]. The estimated healthcare cost of stones in 2007 was $3.79 billion in the United States. Due to population growth, the cost of stone disease could rise by $780 million by 2030 [2]. For this reason, it is important to explore all possible relationships that may lead to the development of stones. 

Nephrolithiasis is universally understood to be a multifactorial disease resulting from genetic and environmental factors including gender, diet, calcium, and uric acid excretion. Notably, several of these factors may be related to body size. Because men are more likely to develop kidney stones and on average have a larger body size, height may be an important risk factor for stone formation.

Several studies have demonstrated that shorter adult height is associated with numerous conditions such as hypertension, hypercholesterolemia, and cardiovascular diseases [3-6]. More importantly, it has been shown that it has been correlated with proteinuria in both type 1 and type 2 diabetes mellitus [7,8]. However, other studies have demonstrated an inverse relationship between height and mortality that was dependent on gender and only within a certain range of height [9]. Similarly, it has been shown that height was not associated with all-cause mortality [10].

Additionally, stones have been shown to be positively correlated with high body mass index (BMI) [11]. This is likely due to dietary factors. Although height is a component of BMI, there is minimal literature regarding the relationship of height to stone prevalence adjusting for weight. Curhan et al. discussed the possible inverse relationship between height and the incidence of stone formation but recommended further investigation [12]. 

Given the large burden of stone disease as mentioned above, we aimed to examine whether short adult height is associated with the development of kidney stones using a population-based cohort of the National Center for Health Statistics. We show a robust positive inverse correlation between height and kidney stones. This relationship is unaffected by confounders such as age and, more importantly, weight.

## Materials and methods

Data was gathered from the CDC’s National Health and Nutrition Examination Survey (NHANES) dataset. Questionnaire data from March 2017 through March 2020 categorized as “Kidney Conditions - Urology” and “Weight History” was collected. The study was approved by the Physician's Journal of Medicine Review Board, Queens, New York, United States (Approval number: 2205F004). Logistic regression analysis was used to determine an association between current height (inches) and if the participant ever had kidney stones, controlling for weight, gender, age, race, educational level, and marital status.

The outcome variable used in this study was the history of ever having a kidney stone. This was assessed as a response of “yes” to the question "Have you ever had kidney stones?". This was coded as Yes = True, and as No = False.

The predictor variable used was height. This was assessed by standard standing measurements conducted by trained health technicians in a mobile examination center. Kidney stone prevalence was determined by the participant's self-reporting. Covariates such as weight, gender, age, race, educational level, and marital status. were then analyzed.

Statistical Analysis

In order to measure the association between the predictor and outcome variables described above, multivariate logistic regression was used to analyze the relationship between height and the history of kidney stones. The analysis included variables such as weight, gender, age, race, educational level, and marital status. Adjusted odds ratios (OR) and confidence intervals (CI) were computed. All analyses were performed using IBM SPSS Statistics for Windows, Version 28.0 (Released 2021; IBM Corp., Armonk, New York, United States).

## Results

The NHANES cycle from 2017 to 2022 had 9208 participants who completed the relevant surveys, of whom 866 reported a history of kidney stones (10.6%) and 8342 did not. The mean age of the participants who did not have a prior occurrence of kidney stones was 50.65±17.80 years old, and the mean age of participants who had a previous occurrence of kidney stones was 55.89 ±15.87 years old. Those with shorter heights had higher odds of reporting a history of stones (OR: 1.017, 95%CI: 1.005-1.028;). This association was found after controlling for covariates such as age, gender, race, education, and weight. In addition, the male gender and Hispanic race had higher odds of reporting a history of stones (OR: 1.43 and 1.073, respectively) (Tables 1, 2).

**Table 1:** Demographic characteristics of adults (aged 20 years and above) NHANES 2017-2022 (N=9,208) NHANES: National Health and Nutrition Examination Survey

Variable	History of Kidney Stones	No History of Kidney Stones
Gender		
Male	464 (10.39%)	4001 (89.61%)
Female	402 (8.48%)	4341 (91.52%)
Race/Origin		
Mexican American	89 (8.45%)	964 (91.55%)
Other Hispanic	102 (10.97%)	828 (89.03%)
White	413 (12.87%)	2797 (87.13%)
Black	144 (5.86%)	2312 (94.14%)
Asian	65 (5.8%)	1055 (94.2%)
Education Level		
Less than 9th grade	66 (9.27%)	646 (90.73%)
9-11th grade	108 (10.39%)	931 (89.61%)
High school graduate/GED or equivalent	188 (8.49%)	2027 (91.51%)
Some college/AA degree	312 (10.5%)	2659 (89.5%)
College graduate or above	191 (8.46%)	2066 (91.54%)
Marital Status		
Married	525 (9.97%)	4743 (90.03%)
Widowed/Divorced/Separated	231 (10.78%)	1911 (89.22%)
Never married	110 (6.15%)	1678 (93.85%)

**Table 2:** Association between height, age, gender, weight, race, and a history of kidney stones OR: odds ratio; CI: confidence interval

Variables	Coefficient	OR	95%CI	p-value
Standing Height (cm)	0.017	1.017	1.005 - 1.028	0.003
Age in years at screening	-0.018	0.982	.978 - .987	< .001
Male gender	0.357	1.43	1.163 - 1.757	< .001
Weight (kg)	-0.009	0.991	0.988 - 0.995	< .001
Hispanic race	0.07	1.073	1.02 - 1.127	0.006

## Discussion

Adult height is a result of both one’s genetic makeup and of childhood environmental factors including illness, nutrition, and socioeconomic circumstances [13-15]. Therefore, height is an important marker of health. In fact, several studies have demonstrated an inverse relationship between adult height and the risk of various causes of death [16,17].

Although evidence that short stature is related to decreased life expectancy is robust, there remain conflicting studies [18,19]. This conflict in literature demonstrates the complex relationship between stature and life expectancy. Because of this conflict, we set out to examine the association between height and the history of kidney stones.

Our results support an inverse relationship between height and the history of kidney stones that is unaffected by gender, age, race, and weight. Shorter adults had higher odds of reporting a history of kidney stones. This novel finding is consistent with previous literature that height is inversely associated with the risk of disease 

This study also strengthens the well-known relationship between BMI and stone formation. Since BMI is calculated as weight divided by height, both higher weights, and shorter heights would be associated with stone formation when calculated into BMI. Our study shows more specifically that short height is related to stone formation independently of weight while still consistent with higher BMIs being associated with stones. 

Also, though these findings are novel, they are consistent with previous literature regarding height and adverse outcomes in kidney disease. Additionally, our analysis suggests that the male gender is associated with a history of stones. This is consistent with previous literature [20]. 

Some important limitations of this study are, firstly, the ultimate reliability of self-reported health data [21], and secondly, the difficulty to assess the true overall risk of stone formation when stone formation can often occur later in life [22]. Our analysis was conducted on a larger population, including young adults who may form stones later in life but have yet to do so.

## Conclusions

Our results suggest that short height is related to the prevalence of kidney stones independent of weight, age, gender, and race. Though this supports previous literature indicating height to be a component of renal disease, we are the first to examine a large population such as NHANES for the relationship between height and kidney stone formation. Therefore, it would be beneficial to have more research done on this particular topic to solidify this association.
